# Educational Needs for Home-Based End-of-Life Hospice Care Among Community Home-Visiting Nurses

**DOI:** 10.3390/healthcare14213707

**Published:** 2026-11-01

**Authors:** Bora Kim, Chul-Gyu Kim, Jung-Ha Son

**Affiliations:** 1 College of Nursing, Chungbuk National University, Cheongju 28644, Republic of Korea; bora0127@chungbuk.ac.kr (B.K.); klecm92@chungbuk.ac.kr (J.-H.S.); 2 Daeshin Medical Clinic, Cheongju 28651, Republic of Korea

**Keywords:** hospice care, terminal care, palliative care, home health nursing, community health nursing, aged, needs assessment

## Abstract

**Highlights:**

**What are the main findings?**

• The perceived importance of home-based end-of-life hospice education exceeded self-reported performance in all 15 domains. The eight highest-priority needs were legal issues in end-of-life care, community linkage, withdrawal of life-sustaining treatment, burnout management, interdisciplinary collaboration, neuropsychiatric symptoms with palliative sedation, philosophical understanding of death, and management of ethical dilemmas.
• In exploratory subgroup comparisons, nurses without advanced practice nurse certification, with less home-based end-of-life care experience, or without a workplace multidisciplinary collaboration system reported the largest importance–performance gaps.

**What are the implications of the main findings?**

• Education could be staged according to home-based end-of-life care experience rather than delivered uniformly, with entry-level content for nurses with less than one year of such experience and advanced content on ethical decision-making, palliative sedation, and care coordination for more experienced nurses.
• Because performance also varied by institution type and the presence of a collaboration system, individual training alone is unlikely to close the gap; institutional collaboration structures and community resource linkages could be developed alongside it.

**Abstract:**

Background/Objectives: Home-based end-of-life care is expanding in Korea with population aging and community care legislation, yet most deaths still occur in institutions, and the educational needs of nurses delivering such care remain poorly characterized. This study prioritized educational needs for home-based end-of-life hospice care (HEHC) among community home-visiting nurses. Methods: In this descriptive cross-sectional study, 120 nurses providing home hospice or end-of-life visiting care in Chungcheong, Korea, recruited through convenience sampling, rated the importance and performance of 56 educational items across 15 domains on 5-point scales. Importance and performance were compared using paired *t*-tests, and differences by participant characteristics using independent *t*-tests and analysis of variance with Scheffé’s post hoc tests. Educational priorities were derived by combining Borich needs scores with the “concentrate here” quadrant of importance–performance analysis. Results: Importance ratings (4.34 ± 0.45) exceeded performance ratings (3.23 ± 0.83) overall (*t* = 11.358, *p* < 0.001; Cohen’s *d*_z_ = 1.04) and in all 15 domains. Eight priorities emerged: legal issues in end-of-life care, linkage with community and external institutions, decision-making on withdrawal of life-sustaining treatment, stress and burnout management, interdisciplinary partnership, management of neuropsychiatric symptoms and palliative sedation, philosophical understanding of death, and management of ethical dilemmas. Nurses without advanced practice nurse certification, without a workplace collaboration system, or with less than one year of home-based end-of-life care experience reported higher importance but lower performance. Conclusions: The observed pattern suggests that education for HEHC could be staged by home-based end-of-life care experience and complemented by institutional collaboration structures and community resource linkages, since individual training alone is unlikely to close the gap.

## 1. Introduction

As of December 2024, adults aged 65 years and older accounted for more than 20% of Korea’s population, and the country was officially classified as a super-aged society [1]. This demographic shift increases the importance of systems that allow older adults and people with chronic illnesses to continue receiving health management and care in their own communities. However, the actual place of death remains concentrated in institutions: 75.1% of deaths in 2024 occurred in medical institutions, whereas only 15.2% occurred at home [2]. By contrast, home was the most frequently preferred place of death (37.7%) in a study of community-dwelling older adults [3]. This discrepancy between the preferred and actual place of death highlights the need to strengthen home-based end-of-life hospice care (HEHC) services so that people can spend their final days with dignity in their chosen setting [2,3]. In response, the Act on Integrated Support for Community Care, including Medical and Nursing Care, took effect on 27 March 2026, requiring medical, long-term, and social care services to be integrated and linked at the community level [4]. Additionally, visiting nurses who meet a minimum experience requirement were included in the nurse staffing standards for home-based hospice institutions, expanding nurses’ role in HEHC [5].

Thus far, Korean home health and visiting nurses have worked mainly in the following contexts: public health center home visit health management programs, long-term care insurance visiting nursing services, and hospital-based home healthcare. Their tasks have centered on chronic disease management, physician-ordered treatments and medication, basic nursing, and health education, and end-of-life (EoL) care has not been established as a core duty [6]. A study involving nurses conducting home visits reported an EoL nursing performance score of only 2.57 out of 4 and found that prior EoL care education directly affected performance, highlighting the need for systematic education [7]. Thus, although these nurses’ role in HEHC is expanding institutionally, their actual EoL care competence has not kept pace.

This role–competence gap is all the more significant given the distinct conditions of home settings. Unlike institutional care, HEHC is delivered in settings where immediate access to health personnel and equipment is difficult; therefore, nurses require a broad competence set extending beyond pain and symptom management to recognizing the dying phase, appropriate communication, psychosocial and spiritual care, ethical decision-making, and multidisciplinary collaboration [8]. This requirement is not specific to Korea: international reviews and qualitative studies of home-based palliative care nursing identify the same broad competence set and report that nurses in these roles frequently describe insufficient preparation, limited organizational support, and a need for systematic education, referral, and linkage systems between institutions [9]. Korean home-based hospice nurses likewise report emotional burden and ethical conflicts while caring for dying patients [10]. Therefore, education for these nurses must cover inter-institutional referral and linkage, ethical decision-making, and stress and burnout management alongside clinical competence.

Evidence to guide the design of such systematic and practical education remains limited. Previous studies on educational needs in hospice and EoL care have targeted the following groups: nursing home staff, including non-nursing personnel [11]; hospice teams in medical institutions comprising clinicians, volunteers, care assistants, social workers, and clergy [12]; nurses, nursing assistants, and care workers in long-term care facilities [13]; and community home-care workers (i.e., care and social workers) [14]. Among hospital hospice teams, the importance–performance gap was largest in spiritual care [12], and among long-term care facility nurses, spiritual, EoL, and bereavement care were identified as educational priorities [13]. Further, community home-care workers reported performance below importance, but their highest-need items clustered around basic care, such as hand hygiene and skin cleanliness, while core EoL items, such as accompanying the dying person and preparing for death, were rated low in importance [14]. Thus, prior work has concentrated on facility- or institution-based staff and non-nursing personnel, and studies examining the educational needs for HEHC among nurses who deliver this care directly in the community remain scarce.

Developing an educational program also requires identifying the content that should be taught first, not merely confirming that education is required. Importance–performance analysis (IPA) places items on a matrix whose axes mark the mean importance and performance of each item, allowing the “concentrate here” region of high importance and low performance to be identified visually [15]. However, because IPA divides items into quadrants using grand means, it cannot rank items within the same quadrant. Meanwhile, the Borich needs assessment computes a needs score by weighting the difference between the required competence level (RCL) and present competence level (PCL) by the mean required level, thereby ranking items quantitatively [16]. However, the Borich model yields only a ranking and provides no criterion for how far down the ranking items should be treated as priorities. To address these constraints, Cho [17] proposed combining Borich needs rankings with the quadrants of the locus for focus model to determine priorities. Because IPA, like the locus for focus model, places items in quadrants using means as reference lines, it can be combined with Borich needs on the same logic. Applying both methods allows items to be ranked by Borich needs while limiting the range of priorities to the IPA “concentrate here” region, yielding educational priorities more systematically. This integrated approach has been employed in Korean nursing education research [18].

Against this background, the current study aimed to examine the importance and performance of educational content for HEHC among nurses providing home hospice or EoL visiting care in the community and to identify educational priorities by applying IPA and Borich needs assessment. Specifically, the study examined (1) participant characteristics; (2) differences in the importance and performance of educational content according to these characteristics; (3) importance, performance, and their differences by domain and item; and (4) the top-priority educational needs derived by combining the Borich needs rankings with IPA. The results could inform the development of educational programs to strengthen nursing competencies in HEHC and the establishment of education and support systems for the community home-care workforce.

## 2. Materials and Methods

### 2.1. Design

This study used a descriptive cross-sectional survey design to examine the importance and performance of educational content for HEHC among nurses providing home hospice or EoL visiting care in the community and to identify educational priorities by applying IPA and Borich needs assessment. This study was conducted in accordance with the STrengthening the Reporting of OBservational studies in Epidemiology (STROBE) statement for cross-sectional studies [19] (File S1).

### 2.2. Participants

Participants were nurses who directly provided home hospice or EoL visiting care in the community. The participants were recruited through convenience sampling from the five types of institution through which home visiting care is delivered in the Chungcheong region of Korea: home healthcare centers, home-visiting nursing agencies, hospital-affiliated home healthcare, public health centers, and long-term care institutions. Eligible participants held nursing licenses, directly provided home hospice or EoL visiting care in the community, understood the study purpose, and voluntarily consented to participate. Nurses whose duties were limited to nursing administration or management without direct home visiting practice were excluded. Convenience sampling was used because no national registry identifies nurses who provide home hospice or EoL visiting care in Korea, and these nurses are dispersed across several institution types and employment arrangements; probability sampling was therefore not feasible.

G*Power 3.1 was used for the sample size calculation [20]. Based on a prior study of educational needs using importance and performance evaluations [21], a two-tailed paired *t*-test with α = 0.05, effect size *d* = 0.25, and power (1 − β) = 0.80 indicated a minimum of 128 participants. During the data collection period, 171 nurses accessed the online survey and 128 submitted a questionnaire (submission rate: 74.9%). Eight of these left 20% or more of the educational needs instrument unanswered, including five who did not respond to the importance scale at all, and were excluded as non-completers; 120 questionnaires were therefore analyzed, eight fewer than the calculated minimum.

### 2.3. Measures

#### 2.3.1. General and Work-Related Characteristics

Based on a previous study [22], the following general and work-related characteristics were assessed: sex, age, educational level, advanced practice nurse (APN) certification, type of institution, employment status, experience at the current workplace, and total clinical experience. Characteristics related to home hospice and EoL visiting care included years of home hospice or EoL visiting care experience and the number of EoL care experiences. Experience variables were recorded in years or as counts and grouped for analysis into the categories shown in Table 1; home hospice or EoL visiting care experience was grouped as <1, 1–2, and ≥3 years, and the number of EoL care experiences as <10, 10–49, and ≥50. These categories were chosen to reflect career stages relevant to the design of staged educational programs. Integrated care characteristics comprised experience in integrated care and types of integrated care activities. Education- and collaboration-related characteristics comprised previous education, the presence of a multidisciplinary collaboration system in the workplace, and collaborating professions. Types of integrated care activities and collaborating professions were surveyed as multiple-response items.

#### 2.3.2. Educational Needs for HEHC

Educational needs were measured using an instrument adapted for home hospice and visiting practice settings from the 78-item hospice and palliative care educational needs instrument developed by Choi et al. [23]. Permission to use and modify the instrument was obtained from its developers. The original instrument comprises 13 domains. Items with low relevance to HEHC or with overlapping content were deleted or merged, and the remaining items were revised and supplemented to reflect the content required in home hospice and visiting settings. These revisions were based on a review of the literature on the competencies required of nurses providing home-based palliative and EoL care [8,9] and on input from the research team and experts in home hospice practice. The resulting home-specific content included differences between home and hospital hospice, criteria for emergency transport and referral, and safety during solo home visits (Table S1). Additional items were developed to address content not covered by the source instrument, and the revised instrument comprised 15 domains compared with the original 13. The content validity of the revised instrument was examined by three hospice APNs and two nursing professors. The item-level content validity index ranged from 0.80 to 1.00, and the scale-level content validity index (average) was 0.993. The final instrument, reflecting expert opinions, comprised 15 domains and 56 items. In this study, the domains were used as content categories to organize and report educational priorities for curriculum planning, rather than as empirically validated subscales. Expert review provided evidence of content relevance and coverage; however, the dimensional structure of the revised instrument was not examined using exploratory or confirmatory factor analysis. Accordingly, construct validity has not been established for the proposed domains. All educational needs indices were calculated at the item level, and domain-level values were descriptive summaries of the corresponding item-level results. The importance and performance of each item were rated on separate 5-point Likert scales, with higher scores indicating higher importance and performance. The questionnaire was administered in Korean, the language of the original instrument and of all participants. In this study, Cronbach’s α was 0.972 for importance and 0.986 for performance.

### 2.4. Data Collection

Data were collected via an online survey from 9 July to 26 July 2026. A study information sheet and a link to the online survey were distributed with the cooperation of the Korean Association of Visiting Nurses, the Korean Academy of Home Care Nursing, and home healthcare centers and public health centers in the Chungcheong region. Nurses who wished to participate reviewed the online information sheet describing the study purpose and methods, the voluntary nature of participation, and the protection of personal information; provided electronic consent; and completed the survey anonymously. As stated in the study information sheet, completing the questionnaire was estimated to require approximately 20 min.

### 2.5. Data Analysis

Data were analyzed using IBM SPSS Statistics for Windows, Version 26.0 (IBM Corp., Armonk, NY, USA) [24]. A total of 120 questionnaires were included in the analysis. Missing responses were replaced with the mean of the observed responses for the corresponding item, separately for importance and performance. Of 13,440 item-level responses (120 participants × 56 items, each rated for importance and performance), 18 (0.13%) were imputed; these were scattered across 16 different items, with no more than two omissions in any single item. As a sensitivity analysis, the domain- and item-level analyses were repeated using complete cases only (*n* = 107); the results are summarized in Section 3.5. General and work-related characteristics, as well as those related to HEHC, integrated care, education, and collaboration, were summarized as frequencies and percentages or means and standard deviations. Multiple-response items were presented as frequencies and percentages. Differences in importance and performance evaluations according to participant characteristics were analyzed using independent *t*-tests for two groups and one-way analysis of variance (ANOVA) for three or more groups, with Scheffé’s post hoc tests when the overall test was significant. Homogeneity of variance was examined using Levene’s test; where this assumption was not met, Welch’s ANOVA with Games–Howell post hoc tests was additionally performed to confirm the results. Differences between importance and performance evaluations were analyzed using paired *t*-tests, with statistical significance set at *p* < 0.05. A Bonferroni correction was applied to the 56 item-level paired comparisons (adjusted α = 0.05/56 = 0.0009), and all comparisons remained statistically significant. No corresponding adjustment was applied to the subgroup comparisons. These were treated as exploratory analyses to describe variation in educational needs across participant characteristics rather than to test prespecified hypotheses, and the sample size was not determined to ensure adequate power for subgroup hypothesis testing. Effect sizes are reported for all comparisons to support interpretation alongside *p*-values.

Educational priorities were assessed using the Borich needs assessment and IPA. For the Borich needs score, the RCL was set as importance and the PCL as performance; the difference between importance and performance for each item was weighted by the mean importance: Borich needs score = Σ[(RCL − PCL) × MCL]/N, where MCL is the mean required competence level for the item and N is the number of participants. Higher Borich needs scores were interpreted as indicating greater educational needs. For IPA, items were allocated to four quadrants using the grand means of importance and performance as reference lines, and the second quadrant (high importance, low performance; “concentrate here”) was categorized as the region for concentrated improvement. Final educational priorities were obtained by combining the items falling in IPA Quadrant II with their Borich needs rankings.

Generative artificial intelligence was not used to generate data or to design, conduct, analyze, or interpret this study.

### 2.6. Ethical Considerations

This study was approved by the Institutional Review Board of Chungbuk National University (IRB No. CBNU-2026-A-0055; approved on 28 April 2026). Through the online study information sheet, participants were informed of the study purpose and methods, the survey content, the protection of personal information, the voluntary nature of participation, and the possibility of withdrawing at any time. The survey was conducted only with participants who provided electronic consent. The survey was anonymous and did not collect directly identifying information (e.g., name or date of birth). Participants were informed that neither their participation nor their responses would disadvantage them and that they could stop at any point during the survey. Contact details for sending a token of appreciation were collected through a separate online form that was not linked to the survey responses. The collected data were coded, stored securely with access restricted to the researchers, and used only for research purposes.

## 3. Results

### 3.1. General and Work-Related Characteristics of Participants

Of the 120 participants, 115 were women (95.8%). The mean age was 45.99 years, and a bachelor’s degree was the most common educational level (57 participants, 47.5%). Forty-nine participants (40.8%) held APN certification. Home-visiting nursing agencies were the most common type of institution (52, 43.3%), and 95 participants (79.2%) were employed full-time. The mean experience at the current workplace was 5.20 years, and the mean total clinical experience was 13.47 years.

The mean home hospice or EoL visiting care experience was 2.79 years, and the mean number of EoL care experiences was 16.57. Eighty-five participants (70.8%) had experience in integrated care, and 97 (80.8%) had previous related education. Sixty participants (50.0%) reported having a multidisciplinary collaboration system in their workplace (Table 1).

### 3.2. Differences in Importance and Performance Evaluations According to Participant Characteristics

Importance evaluations differed significantly by educational level (*F* = 4.767, *p* = 0.010), APN certification (*t* = −4.964, *p* < 0.001), type of institution (*F* = 4.474, *p* = 0.002), experience at the current workplace (*F* = 2.451, *p* = 0.038), home hospice or EoL visiting care experience (*F* = 3.331, *p* = 0.039), number of EoL care experiences (*F* = 4.424, *p* = 0.014), and the presence of a multidisciplinary collaboration system (*t* = −3.616, *p* < 0.001). Importance evaluations did not differ significantly by age, sex, employment status, total clinical experience, or previous education.

In Scheffé’s post hoc tests, importance evaluations were higher in the bachelor’s degree group than in the master’s degree or higher group, higher among those with less than one year of home hospice or EoL visiting care experience than among those with three or more years, and higher among those with fewer than 10 EoL care experiences than among those with 10–49. No significant pairwise differences were identified for type of institution or for experience at the current workplace.

Performance evaluations differed significantly by educational level (*F* = 15.242, *p* < 0.001), APN certification (*t* = 8.773, *p* < 0.001), type of institution (*F* = 13.966, *p* < 0.001), employment status (*t* = 2.988, *p* = 0.004), experience at the current workplace (*F* = 2.518, *p* = 0.033), total clinical experience (*F* = 5.189, *p* < 0.001), home hospice or EoL visiting care experience (*F* = 37.341, *p* < 0.001), number of EoL care experiences (*F* = 13.732, *p* < 0.001), previous education (*t* = 2.956, *p* = 0.004), and the presence of a multidisciplinary collaboration system (*t* = 3.728, *p* < 0.001).

In Scheffé’s post hoc tests, performance evaluations were higher in the master’s degree or higher group than in the associate and bachelor’s degree groups. By institution type, performance evaluations were lower in public health centers than in all other institution types, and higher in home healthcare centers and in hospital-affiliated home healthcare than in visiting nursing agencies. For total clinical experience, performance evaluations were higher in the 5–9-year, 10–14-year, and ≥20-year groups than in the <5-year group. For home hospice or EoL visiting care experience, they were higher in the 1–2-year and ≥3-year groups than in the <1-year group. For the number of EoL care experiences, they were higher in the 10–49 group than in the <10 group (Table 2).

### 3.3. Importance and Performance Evaluations and Borich Needs by Educational Domain

The overall importance value was 4.34 ± 0.45, and the overall performance value was 3.23 ± 0.83, with the former significantly higher (*t* = 11.358, *p* < 0.001; Cohen’s *d*_z_ = 1.04, 95% CI of the gap [0.916, 1.303]). Importance evaluations were significantly higher than performance evaluations in all 15 educational domains (*p* < 0.001), with paired effect sizes ranging from *d*_z_ = 0.69 (infection prevention and safety management) to 1.13 (pediatric hospice care) (Table 3).

The domain with the highest importance was pain and symptom palliation (4.46 ± 0.52), followed by care of patients with non-cancer terminal illness (4.44 ± 0.60) and emergency assessment and referral (4.44 ± 0.57). Performance was highest for care of patients with non-cancer terminal illness (3.59 ± 0.94), followed by infection prevention and safety management (3.55 ± 0.92).

By domain, Borich needs were highest for pediatric hospice care (6.527), followed by the development of a hospice collaboration system (5.761), complementary and alternative therapies (5.389), ethics and law in hospice care (5.216), and understanding hospice and palliative care (5.170) (Table 3).

### 3.4. Borich Needs by Educational Item

Among the 56 educational items, the highest Borich needs score was for “children’s understanding of death” (6.662), followed by “care methods for children and their families” (6.392); “legal issues, including wills, inheritance management, and organ transplantation” (6.193); “strategies for linkage and partnership with community and external institutions” (6.046); and “decision-making related to withdrawal of life-sustaining treatment” (5.775).

These items were followed by “stress and burnout management” (5.616), “manipulative and body-based therapies, including massage and acupressure” (5.534), “partnership with professionals from other disciplines” (5.476), “current status and future directions of hospice and palliative care in Korea and abroad” (5.408), and “management of neuropsychiatric symptoms and palliative sedation” (5.370) (Table S1).

### 3.5. Final Educational Priorities from IPA and Borich Needs

**Figure 1:**
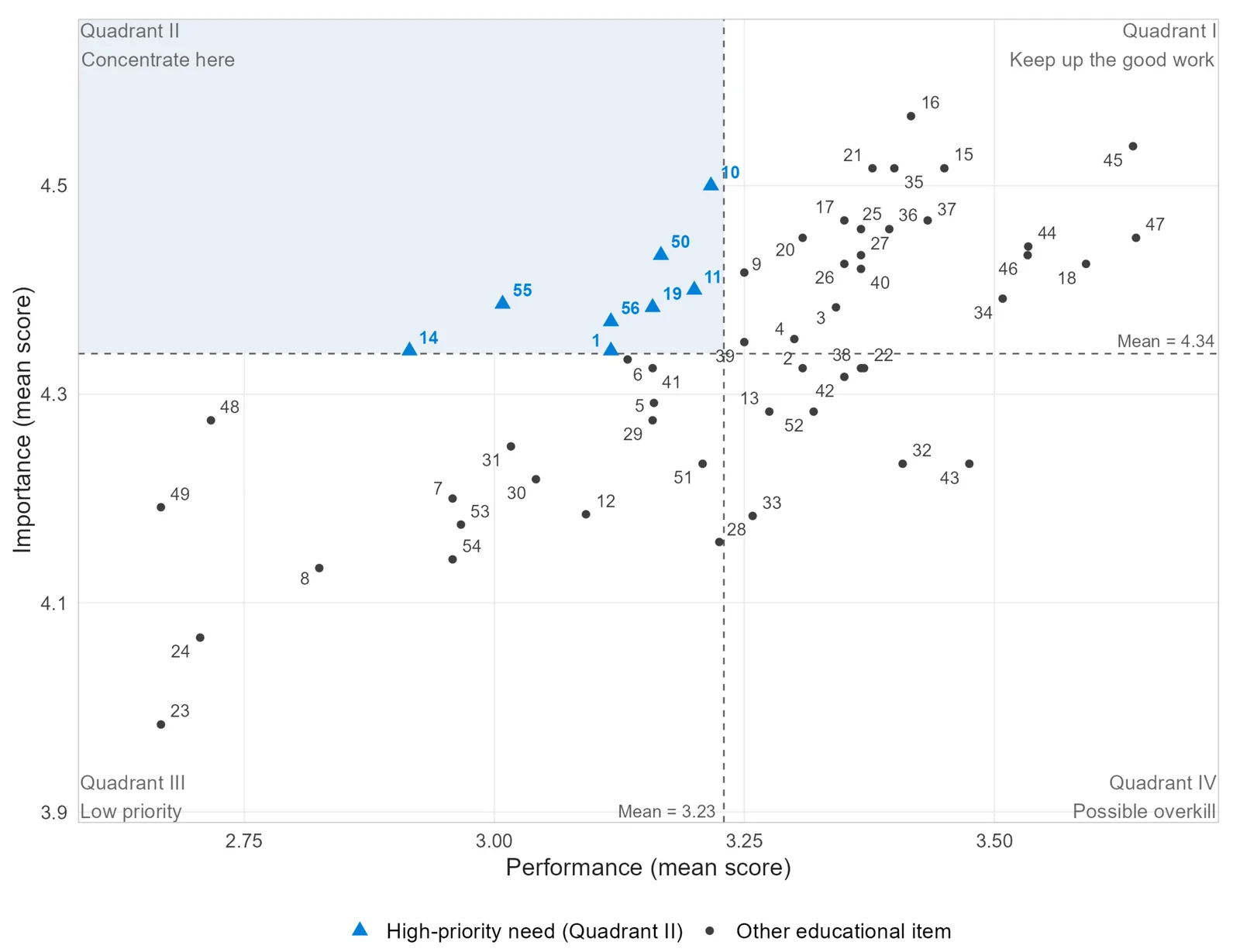
Importance–performance analysis (IPA) matrix of educational needs for home-based end-of-life hospice care (*N* = 120). Reference lines indicate the grand means of performance (3.23) and importance (4.34). Items in Quadrant II (“concentrate here”) were 1 (philosophical and stage-based understanding of death), 10 (decision-making related to withdrawal of life-sustaining treatment), 11 (management of situations involving ethical dilemma), 14 (legal issues, including wills, inheritance management, and organ transplantation), 19 (management of neuropsychiatric symptoms and palliative sedation), 50 (stress and burnout management), 55 (strategies for linkage and partnership with community and external institutions), 56 (partnership with professionals from other disciplines). The item numbers correspond to those in Table S1.

Items were allocated to four quadrants using the grand means of importance and performance, and Quadrant II (high importance, low performance; “concentrate here”) was classified as the region for concentrated improvement. Combining the items in IPA Quadrant II with their Borich needs rankings from Table S1 yielded eight final educational priorities. In order of Borich needs these were “legal issues, including wills, inheritance management, and organ transplantation,” “strategies for linkage and partnership with community and external institutions,” “decision-making related to withdrawal of life-sustaining treatment,” “stress and burnout management,” “partnership with professionals from other disciplines,” “management of neuropsychiatric symptoms and palliative sedation,” “philosophical and stage-based understanding of death,” and “management of situations involving ethical dilemma” (Figure 1). Two of these items cleared the importance reference line by a very small margin: both “legal issues” and “philosophical and stage-based understanding of death” had a mean importance of 4.342 against a grand mean of 4.339, so their quadrant assignment should be read as borderline rather than firm. A sensitivity analysis restricted to complete cases (*n* = 107) left the domain-level ranking unchanged, with pediatric hospice care and the development of a hospice collaboration system again ranked first and second, and reproduced five of the eight item-level priorities: community and external institution linkage, management of neuropsychiatric symptoms and palliative sedation, partnership with professionals from other disciplines, stress and burnout management, and philosophical and stage-based understanding of death. Each of the three that did not reproduce lay within 0.03 of a reference line: “legal issues, including wills, inheritance management, and organ transplantation” fell just below the importance line, while “decision-making related to withdrawal of life-sustaining treatment” and “management of situations involving ethical dilemma” rose just above the performance line.

## 4. Discussion

This study examined the importance and performance of educational content for HEHC among nurses providing home hospice or EoL visiting care in the community and identified educational priorities by integrating IPA and the Borich needs assessment. Importance ratings significantly exceeded performance ratings overall and in all 15 educational domains. The same direction has been reported for community home-care workers [14], for nurse managers across all competence items [25], and for home care nurses, whose palliative care competence and self-efficacy were insufficient [26]. Because this gap was uniform across domains, the discussion below concentrates on where it is widest and on what distinguishes the groups and settings in which it occurs, rather than restating it domain by domain.

Almost all participants were women, reflecting the composition of the Korean nursing workforce, in which women accounted for 94.9% of nurses working in healthcare institutions in 2020 [27], and, particularly, of community home-visiting nursing, in which women made up 97.5% of 360 home-visiting nurses in a recent Korean survey [7]; the present findings therefore describe a workforce that is predominantly female rather than one balanced by sex. Importance and performance evaluations differed significantly by most of the participant characteristics examined (Table 2). Performance evaluations were higher among certified APNs than among non-certified nurses and increased with educational level, consistent with reports that advance care planning education improved APN students’ knowledge and perceptions of the nurse’s role in EoL treatment decisions [28], and that EoL care preparedness and educational priorities differed by occupational group among long-term care facility nursing staff [13]. Importance ran in the opposite direction, however, being higher among nurses without APN certification and among those with a bachelor’s rather than a master’s degree or higher—possibly because nurses who repeatedly encounter competences they lack perceive the need more acutely. Both ratings were self-reported, so the higher performance scores of certified and more highly educated nurses may reflect greater confidence in their own practice as much as demonstrably greater competence, just as the higher importance ratings of less-qualified nurses may reflect sharper awareness of their own gaps. What these comparisons establish is a difference in perceived preparedness; whether it corresponds to a difference in actual capability would require observational or performance-based assessment.

Performance evaluations were lowest among nurses with the least total clinical experience, and the pattern was clearer for home hospice or EoL visiting care experience: those with less than one year rated importance high but performance low, whereas performance was markedly higher in the more experienced groups. Nurses with fewer EoL care experiences showed the same pattern. Shimizu et al. [29] and Watanabe et al. [30] similarly reported greater self-reported performance and confidence, and fewer difficulties, among home palliative care nurses with more experience of caring for dying patients at home. In this study, however, total clinical experience was related to performance but not to importance, whereas home hospice or EoL visiting care experience and the number of EoL care experiences were related to both, and the former accounted for more than twice the variance in performance explained by total clinical experience (η^2^ = 0.390 versus 0.153). Competence in HEHC may therefore relate more specifically to EoL care experience in a home setting than to the accumulation of general clinical experience. Nurses with less than one year of such experience, whose performance was significantly lower than that of both more experienced groups (Table 2), are accordingly referred to as less experienced in this study, and it is this variable that underlies the staged education proposed below. The number of EoL care experiences was not monotonic: performance peaked in the intermediate group and fell again among nurses with the most experiences, in whom importance evaluations were also higher, possibly reflecting the emotional burden of repeated EoL care [10,31]. As that group comprised only 13 participants, this finding should be interpreted with caution.

Previous education was associated with a difference in performance only, signifying that the content was rated as important regardless of prior education. Lähteenmäki et al. [32] reported improvements in symptom management, teamwork, and interpersonal competence after specialist palliative care education, even among nurses with little palliative care experience; as that study used a pretest–posttest design whereas the present study was cross-sectional; however, the relationship observed here cannot be interpreted as an effect of education. These findings nonetheless indicate where current educational provision falls short. Continuing education for Korean home-visiting nurses has centered on chronic disease management, prescribed treatments, and basic nursing rather than EoL care [6], and their formal role in home-based hospice has been established only recently [5]. Rather than requiring a separate curriculum, the priorities identified here could be incorporated into the continuing professional development already provided to this workforce, with entry-level and advanced modules differentiated by home EoL care experience.

Differences in work environment were equally marked. Performance evaluations were lowest in public health centers (2.12) and highest in hospital-affiliated home healthcare (3.91, versus 3.00 in home-visiting nursing agencies), and were higher among full-time than among part-time nurses (3.32 versus 2.88). Public health center home visits focus on chronic disease management and health education, limiting opportunities to deliver EoL care, whereas hospital-affiliated agencies work within institutional linkages that provide more contact with terminally ill patients. These differences may reflect the mandate and caseload of each institution rather than the preparedness of the individual nurses employed there, so the low performance recorded in public health centers is better read as limited opportunity to practice than as an individual competency deficit.

The presence of a multidisciplinary collaboration system in the workplace showed the same pattern: performance evaluations were higher where a system existed (3.50 versus 2.96), whereas importance evaluations were higher where it did not (4.48 versus 4.20). A systematic review by Anita et al. [33] identified insufficient team coordination, inadequate organizational support, and a lack of education as major barriers for home palliative care nurses. Low ratings on collaboration and linkage content should therefore be interpreted with care. They may signal an educational deficit, but they may equally reflect structural barriers—absent referral pathways, limited integration between home care and hospice or hospital services, and institutional constraints on the time and authority a visiting nurse has to coordinate care—under which the competence cannot be exercised even when it has been taught. The present data cannot separate these explanations, because nurses rated what they do rather than what they could do within a supportive structure. Education targeting these areas may therefore be useful but may not be sufficient on its own, and would need to be paired with the service integration and organizational arrangements that make collaboration and community resource linkage practicable.

The domain with the highest importance in this study was pain and symptom palliation, followed by care of patients with non-cancer terminal illness and emergency assessment and referral. Performance in these domains was also comparatively high, at 3.38, 3.59, and 3.36, all above the overall mean of 3.23, so their domain-level Borich needs ranked only 7th, 14th, and 8th of 15. Pain and symptom management is presumably the most basic clinical competence in EoL care and is performed relatively frequently; a scoping review by Ghaffarifar et al. [8] similarly identified physical symptom management, medication management, and care in the dying phase as core competencies of home-based palliative care nurses, and studies of home EoL care in Korea and abroad report that physical symptom management needs increase as the dying phase progresses [34,35]. Domain-level performance did not hold for every item within the domain, however: of its eight items, only management of neuropsychiatric symptoms and palliative sedation fell in the IPA “concentrate here” region. Education in this domain could therefore concentrate on the items whose performance remains relatively low rather than on general symptom control.

In the combined IPA and Borich results, strategies for linkage and partnership with the community and external institutions emerged as the second highest educational priority, and partnership with professionals from other disciplines was also among the priorities; the domain to which both items belong—development of a hospice collaboration system—ranked second of 15 in Borich needs, at 5.761. Performance on the community and external institution linkage item was 3.01, the second lowest of the eight priority items. In exploratory subgroup comparisons, this pattern was consistent with the higher performance observed among nurses within a multidisciplinary collaboration system, and the higher importance ratings among nurses without one suggest that the need for linkage competence is perceived most acutely where the structure is absent. Anita et al. [33] described nurses in home palliative care as coordinators connecting various professionals with patients and families, noting that clear roles and organizational support are needed for collaboration. Rather than simply conveying a list of community resources, therefore, education could be structured around actual cases: worked referral scenarios in which nurses apply transfer and consultation criteria to a deteriorating patient, structured handover and information-sharing formats, and joint case conferences with the partner institutions a given agency actually works with. These formats would allow nurses to rehearse referral criteria, case management, and care coordination with physicians, social workers, hospice institutions, emergency medical institutions, and long-term care institutions within the collaboration structure in which they must be used.

Decision-making regarding the withdrawal of life-sustaining treatment and the management of situations involving ethical dilemma were also high priorities. Jo et al. [28] found that knowledge, confidence in supporting patients’ advance decisions, and perception of the nurse’s role in EoL treatment decisions improved significantly among Korean APN students after advance care planning education, and Svendsen et al. [36] reported that home palliative care nurses experience conflicts between patients’ values and guidelines and find it difficult to clarify their role in shared EoL decision-making. Education for HEHC could therefore include not only knowledge of the laws and systems governing life-sustaining treatment but also structured case deliberation with an explicit framework for weighing patient values, family positions, and legal constraints, together with rehearsed conversations for the situations nurses meet most often: a family request to stop treatment at home, disagreement between patient and family about transfer, and documenting an advance decision.

The domain “ethics and law in hospice care” ranked fourth of 15 in domain-level Borich needs at 5.216, and at the item level, “legal issues, including wills, inheritance management, and organ transplantation” ranked third of 56 items at 6.193 and became the highest of the eight priorities once the IPA quadrant was applied, with the lowest performance of any priority item (2.92). Nurses working in home settings cannot themselves resolve matters such as wills or inheritance, so this result is better read as a need for orientation and referral than for legal training: content on law and institutional systems may be addressed more realistically through guidelines available when cases arise, together with referral pathways to legal and social work consultation, than through universal mandatory education. Its mean importance (4.342) nonetheless exceeded the grand mean (4.339) by a very small margin, so its position among the priorities should be treated as borderline. Philosophical and stage-based understanding of death entered the priority set on the same narrow margin. It is foundational content in standard hospice curricula rather than an advanced competency, and its appearance here is consistent with the finding that continuing education for this workforce has centered on chronic disease management rather than end-of-life care; it therefore points to a gap in basic preparation rather than to a specialized training need.

Stress and burnout management was among the highest priorities, showing that care for nurses themselves is also an important educational domain for those who continuously provide home hospice or EoL visiting care. Bang et al. [10] found that Korean home-based hospice nurses experienced emotional burden and psychological difficulties during repeated EoL care, and in a meta-analysis by Gómez-Urquiza et al. [31], emotional exhaustion was reported by 24% of palliative care nurses, depersonalization by 30%, and low personal accomplishment by 28%, with work-related factors as major contributors. Rather than treating self-care as an individual responsibility, therefore, educational systems could incorporate organizational support: a brief self-screening measure of emotional exhaustion administered at set intervals, debriefing scheduled routinely after a patient’s death rather than offered on request, peer support, and a named referral route to counseling that does not require a nurse to disclose distress to a supervisor first.

The management of neuropsychiatric symptoms and palliative sedation was also among the top priorities. Palliative sedation may relieve refractory suffering, but it requires judgment of indications, monitoring of the patient’s condition, ethical decision-making, and communication with the family. Zuleta-Benjumea et al. [37] reported that nurses performing palliative sedation held considerable knowledge but had acquired it largely through practical experience, and that a lack of formal education affected their confidence and emotional burden. Given that performance in this study differed by home EoL care experience, education on this content would be better delivered through cases of delirium, anxiety, agitation, and refractory symptoms that support both clinical judgment and communication than through lectures alone.

By contrast, pediatric hospice care ranked highest among domains in the Borich needs assessment, and at the item level, “children’s understanding of death” and “care methods for children and their families” ranked first and second; yet neither item fell in the IPA “concentrate here” region, and both were excluded from the final priorities. This divergence reflects the distinct functions of the two methods rather than an inconsistency in the findings. The Borich score ranks content according to the importance-weighted gap between required and current competence. Thus, content may rank highly when reported performance is low, even if its importance rating does not exceed the overall importance mean. In contrast, IPA classifies an item as a “concentrate here” priority only when importance exceeds the grand mean and performance falls below the grand mean. Combining the two methods therefore identifies content that was both highly ranked by needs score and perceived by the study sample as important but insufficiently performed. Content ranked highly by Borich analysis alone is retained as a candidate for targeted rather than universal education. Pediatric hospice care is such a case, as are complementary and alternative therapies (Borich needs = 5.389, third) and digital-based home hospice care (Borich needs = 4.973, sixth). These domains had low reported performance and importance ratings below the overall mean. Schröder et al. [38] found that home-based pediatric palliative care requires relationship building with children and parents, a multidisciplinary team, and sufficient skilled personnel and resources. Pediatric hospice care may therefore be better structured as advanced or elective education for nurses caring for EoL pediatric patients than as uniform education for the entire home-care workforce, while the other two domains have not yet been established as standard work in home EoL care practice.

### Strengths and Limitations

Several study limitations should be noted. First, participants were recruited through convenience sampling from five types of community home visiting care setting in the Chungcheong region, and 95.8% were women; the sample is therefore not representative of the national home-visiting nursing workforce, and the findings should be read as describing the educational needs of this sample rather than as estimates for Korean home hospice nurses generally. Second, data were collected through a self-administered online survey distributed indirectly by professional organizations and institutions. Because the researchers had no access to the sampling frame and could not determine how many nurses received the link, a true response rate could not be calculated. Additionally, nurses with a stronger interest in EoL education may have been more likely to respond. Third, both importance and performance were self-reported rather than observed in practice and may therefore differ from actual competence; self-assessment is especially prone to bias because those with lower competence tend to overestimate their ability [39]. The subgroup differences reported here are accordingly differences in perceived preparedness. Fourth, importance was rated uniformly high (4.34 ± 0.45 overall), and this possible ceiling effect may have reduced variability and limited the capacity to discriminate among items on the importance axis, which defines one boundary of the IPA quadrants; 15 of the 56 items lay within 0.05 of the importance grand mean, so the quadrant assignment of items in that band is correspondingly unstable. Fifth, as the study involved a cross-sectional survey, the relationships between participant characteristics and importance or performance cannot be interpreted causally. Sixth, the 18 item-level omissions that remained after the exclusion described in the next limitation were replaced by the corresponding item mean. Although the proportion was small—0.13% of responses, with no single item carrying more than two—mean imputation may reduce variance or influence some comparisons. The complete-case sensitivity analysis is therefore reported in Section 3.5. Seventh, eight respondents who left 20% or more of the instrument unanswered were excluded as non-completers. They were older than the analyzed sample (mean 57.0 versus 46.0 years), less likely to hold APN certification, more often employed in public health centers, and more often working without a multidisciplinary collaboration system; the excluded group therefore resembles participants who reported larger importance–performance gaps in the exploratory subgroup comparisons, and its educational needs are not represented in these results. Eighth, only the content validity of the revised instrument was assessed through expert review; its 15-domain structure was not empirically examined using factor analysis. The very high Cronbach’s α coefficients may reflect item redundancy and do not establish unidimensionality or construct validity. Accordingly, the domains should be interpreted as content categories rather than validated subscales. Further research is needed to evaluate the instrument’s dimensional structure and potential item redundancy. Ninth, the subgroup comparisons in Table 2 were not adjusted for multiple testing; with each participant characteristic examined for both importance and performance, some significant results may have arisen by chance, and those comparisons are exploratory.

Notwithstanding these limitations, this study has several strengths. First, to our knowledge, it is among the first studies to assess educational needs for HEHC among nurses who directly provide home hospice or EoL visiting care in the community, a workforce whose formal role in home-based hospice was established only recently [5] and whose needs have rarely been examined separately from those of facility-based staff or non-nursing personnel [11,12,13,14]. Second, participants came from five types of institution through which home visiting care is delivered, allowing differences in work environment and collaboration structure to be examined alongside individual characteristics. Third, the instrument was adapted for home settings from an established hospice and palliative care curriculum [23] and showed high content validity indices. Fourth, integrating IPA with the Borich needs assessment allowed items to be ranked by need while restricting priorities to items with both high importance and low performance; this prevented domains with low performance but comparatively low importance, such as pediatric hospice care and complementary and alternative therapies, from being treated as universal priorities. Finally, the item-level results (Table S1) provide a concrete basis for selecting content when educational programs are designed.

## 5. Conclusions

Importance evaluations of educational content for HEHC exceeded performance evaluations in all 15 domains, and the highest educational priorities were legal issues in end-of-life care, linkage with community and external institutions, decision-making on withdrawal of life-sustaining treatment, stress and burnout management, interdisciplinary collaboration, management of neuropsychiatric symptoms and palliative sedation, philosophical understanding of death, and management of ethical dilemmas. In exploratory subgroup comparisons, nurses without APN certification, with less home EoL care experience, or without a workplace collaboration system reported the largest importance–performance gaps. On this basis, education for HEHC would be better staged than delivered uniformly: entry-level content for nurses with less than one year of home EoL care experience covering recognition of the dying phase, symptom and emergency response, and family communication, and advanced content for more experienced nurses covering complex ethical decision-making, palliative sedation, multidisciplinary coordination, and community linkage. Because performance also varied by institution type and the presence of a collaboration system, individual education alone is unlikely to close the gap; agencies could additionally establish referral and consultation pathways with hospice institutions, physicians, and social workers, pair nurses new to home EoL care with experienced colleagues, and hold regular case debriefings after patient deaths. These staged and organizational proposals are practice-oriented inferences drawn from a pattern of perceived needs in one regional sample; they were not evaluated in this study, and both their applicability to the wider Korean home visiting workforce and their effectiveness remain to be tested. Future research should develop and evaluate programs reflecting these priorities, reassess needs across a wider range of regions and institutions, and use qualitative or mixed-methods designs to explore how nurses experience these priority areas in practice.

## Figures and Tables

**Table 1:** General and work-related characteristics of participants (*N* = 120).

Variables	Categories	*n* (%) or *M* ± SD
Sex	Male	5 (4.2)
	Female	115 (95.8)
Age (years)	<40	36 (30.0)
	40–49	44 (36.7)
	≥50	40 (33.3)
	Mean ± SD	45.99 ± 9.42
Education level	Associate degree	24 (20.0)
	Bachelor’s degree	57 (47.5)
	Master’s degree or higher	39 (32.5)
Advanced practice nurse certification	Yes	49 (40.8)
	No	71 (59.2)
Type of institution	Home healthcare center	28 (23.3)
	Home-visiting nursing agency	52 (43.3)
	Hospital-affiliated home healthcare	20 (16.7)
	Public health center/public institution	10 (8.3)
	Long-term care institution	10 (8.3)
Employment status	Full-time	95 (79.2)
	Part-time	25 (20.8)
Experience at current workplace (years)	<1	12 (10.0)
	1–2	30 (25.0)
	3–4	29 (24.2)
	5–6	18 (15.0)
	7–9	18 (15.0)
	≥10	13 (10.8)
	Mean ± SD	5.20 ± 4.36
Total clinical experience (years)	<5	12 (10.0)
	5–9	22 (18.3)
	10–14	37 (30.8)
	15–19	23 (19.2)
	≥20	26 (21.7)
	Mean ± SD	13.47 ± 7.24
Home hospice/end-of-life visiting care experience (years)	<1	41 (34.2)
	1–2	27 (22.5)
	≥3	52 (43.3)
	Mean ± SD	2.79 ± 3.14
Number of end-of-life care experiences	<10	62 (51.7)
	10–49	45 (37.5)
	≥50	13 (10.8)
	Mean ± SD	16.57 ± 25.11
Experience in integrated care	Yes	85 (70.8)
	No	35 (29.2)
Integrated care activities *	Case management	49 (57.6)
	Home visiting healthcare	80 (94.1)
	Service linkage and coordination	31 (36.5)
	Multidisciplinary collaboration	20 (23.5)
Previous education	Yes	97 (80.8)
	No	23 (19.2)
Multidisciplinary collaboration system in the workplace	Yes	60 (50.0)
	No	60 (50.0)
Collaborating professions *	Physician	46 (76.7)
	Social worker	48 (80.0)
	Physical/occupational therapist	28 (46.7)
	Dietitian	16 (26.7)
	Care worker	48 (80.0)
	Wound care specialist nurse	9 (15.0)
	Psychological counselor	12 (20.0)
	Clergy/spiritual care provider	4 (6.7)

Values are presented as *n* (%) or mean ± standard deviation. *M* = mean; SD = standard deviation. * Multiple responses were allowed; therefore, the percentages may exceed 100%. No inferential comparisons were performed for the multiple response variables. Percentages for integrated care activities are based on the 85 participants with integrated care experience. Percentages for collaborating professions are based on the 60 participants reporting a multidisciplinary collaboration system.

**Table 2:** Differences in importance and performance scores according to participant characteristics (*N* = 120).

Variables	Categories	Importance *M* ± SD	*t* or *F*	*p* (Scheffé)	Effect Size	Performance *M* ± SD	*t* or *F*	*p* (Scheffé)	Effect Size
Sex	Male	4.46 ± 0.90	0.314	0.769	0.28	3.24 ± 0.51	0.027	0.979	0.01
	Female	4.33 ± 0.43				3.23 ± 0.84			
Age (years)	<40	4.29 ± 0.52	2.350	0.100	0.039	3.32 ± 0.89	2.730	0.069	0.045
	40–49	4.26 ± 0.38				3.38 ± 0.83			
	≥50	4.46 ± 0.43				2.99 ± 0.74			
Education level	Associate degree ^a^	4.37 ± 0.42	4.767	0.010 (b > c)	0.075	2.75 ± 0.72	15.242	<0.001 (c > a, b)	0.207
	Bachelor’s degree ^b^	4.44 ± 0.45				3.08 ± 0.83			
	Master’s degree or higher ^c^	4.17 ± 0.42				3.74 ± 0.61			
Advanced practice nurse certification	Yes	4.12 ± 0.43	−4.964	<0.001	−0.92	3.83 ± 0.53	8.773	<0.001	1.53
	No	4.49 ± 0.40				2.81 ± 0.74			
Type of institution	Home healthcare center ^a^	4.14 ± 0.38	4.474	0.002	0.135	3.52 ± 0.68	13.966	<0.001 (a, c > b; a, b, c, e > d)	0.327
	Home-visiting nursing agency ^b^	4.44 ± 0.38				3.00 ± 0.74			
	Hospital-affiliated home healthcare ^c^	4.14 ± 0.57				3.91 ± 0.49			
	Public health center/public institution ^d^	4.58 ± 0.39				2.12 ± 0.94			
	Long-term care institution ^e^	4.50 ± 0.49				3.36 ± 0.51			
Employment status	Full-time	4.31 ± 0.41	−1.044	0.304	−0.28	3.32 ± 0.86	2.988	0.004	0.54
	Part-time	4.44 ± 0.56				2.88 ± 0.58			
Experience at current workplace (years)	<1	4.53 ± 0.32	2.451	0.038	0.097	2.80 ± 0.64	2.518	0.033	0.099
	1–2	4.39 ± 0.50				3.31 ± 0.91			
	3–4	4.28 ± 0.38				3.43 ± 0.77			
	5–6	4.34 ± 0.45				3.52 ± 0.76			
	7–9	4.08 ± 0.46				3.13 ± 0.77			
	≥10	4.53 ± 0.43				2.76 ± 0.87			
Total clinical experience (years)	<5 ^a^	4.49 ± 0.33	0.767	0.549	0.026	2.32 ± 0.95	5.189	<0.001 (b, c, e > a)	0.153
	5–9 ^b^	4.24 ± 0.49				3.20 ± 0.76			
	10–14 ^c^	4.30 ± 0.48				3.45 ± 0.74			
	15–19 ^d^	4.35 ± 0.42				3.17 ± 0.83			
	≥20 ^e^	4.40 ± 0.44				3.41 ± 0.71			
Home hospice/end-of-life visiting care experience (years)	<1 ^a^	4.47 ± 0.42	3.331	0.039 (a > c)	0.054	2.52 ± 0.80	37.341	<0.001 (b, c > a)	0.390
	1–2 ^b^	4.35 ± 0.50				3.67 ± 0.59			
	≥3 ^c^	4.23 ± 0.42				3.57 ± 0.55			
Number of end-of-life care experiences	<10 ^a^	4.43 ± 0.38	4.424	0.014 (a > b)	0.070	2.90 ± 0.91	13.732	<0.001 (b > a)	0.190
	10–49 ^b^	4.19 ± 0.44				3.67 ± 0.53			
	≥50 ^c^	4.45 ± 0.66				3.27 ± 0.55			
Previous education	Yes	4.33 ± 0.46	−0.330	0.742	−0.08	3.34 ± 0.80	2.956	0.004	0.69
	No	4.37 ± 0.41				2.78 ± 0.84			
Multidisciplinary collaboration system in the workplace	Yes	4.20 ± 0.42	−3.616	<0.001	−0.66	3.50 ± 0.74	3.728	<0.001	0.68
	No	4.48 ± 0.44				2.96 ± 0.83			

Values are presented as mean ± standard deviation. *M* = mean; SD = standard deviation; *t* = independent *t*-test; *F* = one-way ANOVA. Superscript letters (a, b, c, …) denote the category groups used in Scheffé’s post hoc comparisons; the notation in parentheses reports significant pairwise differences. Effect size is Cohen’s *d* for two-group comparisons and η^2^ for one-way ANOVA (η^2^ = df1*F*/(df1*F* + df2)). Conventional benchmarks: *d* = 0.20/0.50/0.80 and η^2^ = 0.01/0.06/0.14 for small/medium/large effects. No significant pairwise differences were detected by Scheffé’s post hoc tests for type of institution (importance) or for experience at the current workplace (importance and performance), despite significant overall *F* values. Levene’s test indicated unequal variances for importance and performance by the number of end-of-life care experiences (*p* = 0.045 and *p* = 0.001); Welch’s ANOVA (*F* = 4.503, *p* = 0.019; and *F* = 15.476, *p* < 0.001) and Games–Howell post hoc tests yielded the same conclusions. Where Levene’s test indicated unequal variances in a two-group comparison (sex and employment status for importance; advanced practice nurse certification and employment status for performance), Welch’s *t*-test is reported.

**Table 3:** Importance and performance evaluations and Borich educational needs by domain (*N* = 120).

Domain	Educational Domain	Item no.	Importance *M* ± SD	Performance *M* ± SD	Gap	*t*	*p*	d_z_	95% CI of Gap	Borich Needs	Rank
A	Understanding Life and Death	1–4	4.35 ± 0.56	3.27 ± 0.97	1.084	9.737	<0.001	0.89	[0.864, 1.305]	4.716	9
B	Understanding Hospice and Palliative Care	5–8	4.24 ± 0.58	3.02 ± 1.07	1.221	10.120	<0.001	0.92	[0.982, 1.459]	5.170	5
C	Ethics and Law in Hospice Care	9–14	4.35 ± 0.51	3.16 ± 0.87	1.196	11.338	<0.001	1.04	[0.987, 1.405]	5.216	4
D	Pain and Symptom Palliation	15–22	4.46 ± 0.52	3.38 ± 0.90	1.078	10.354	<0.001	0.95	[0.872, 1.285]	4.809	7
E	Complementary and Alternative Therapies	23–24	4.03 ± 0.72	2.69 ± 0.99	1.339	11.811	<0.001	1.08	[1.114, 1.563]	5.389	3
F	Emergency Assessment and Referral	25–27	4.44 ± 0.57	3.36 ± 1.03	1.078	9.566	<0.001	0.87	[0.855, 1.301]	4.784	8
G	Psychosocial and Spiritual Care and Communication	28–35	4.28 ± 0.51	3.25 ± 0.97	1.026	9.654	<0.001	0.88	[0.816, 1.237]	4.393	13
H	Family Care	36–39	4.40 ± 0.57	3.36 ± 0.95	1.039	9.384	<0.001	0.86	[0.820, 1.258]	4.572	12
I	Home Hospice Operation and Management	40–42	4.35 ± 0.54	3.29 ± 0.97	1.062	10.179	<0.001	0.93	[0.856, 1.269]	4.625	11
J	Infection Prevention and Safety Management	43–45	4.40 ± 0.61	3.55 ± 0.92	0.855	7.524	<0.001	0.69	[0.630, 1.080]	3.774	15
K	Care of Patients with Non-Cancer Terminal Illness	46–47	4.44 ± 0.60	3.59 ± 0.94	0.854	8.180	<0.001	0.75	[0.647, 1.061]	3.794	14
L	Pediatric Hospice Care	48–49	4.23 ± 0.69	2.69 ± 1.07	1.542	12.384	<0.001	1.13	[1.295, 1.788]	6.527	1
M	Professional Development of Hospice Team Members	50–52	4.32 ± 0.58	3.23 ± 1.01	1.085	10.236	<0.001	0.93	[0.875, 1.295]	4.695	10
N	Digital-Based Home Hospice Care	53–54	4.16 ± 0.72	2.96 ± 1.17	1.196	9.053	<0.001	0.83	[0.934, 1.457]	4.973	6
O	Development of a Hospice Collaboration System	55–56	4.38 ± 0.59	3.06 ± 1.14	1.316	11.225	<0.001	1.02	[1.084, 1.548]	5.761	2
	Overall	1–56	4.34 ± 0.45	3.23 ± 0.83	1.109	11.358	<0.001	1.04	[0.916, 1.303]	4.808	-

Gap = importance–performance. Paired-samples *t*-tests were performed to compare importance and performance evaluations; all comparisons were considered significant at *p* < 0.001. Borich needs score = [Σ(RCL − PCL) × MCL]/*N*, where RCL = required competence level (importance), PCL = present competence level (performance), MCL = mean of RCL, and *N* = number of participants. dz = Cohen’s d for paired samples (t/√N); 95% CI of gap = mean difference ± t(0.975, 119) × standard error. The conventional benchmarks for *d*_z_ are 0.20/0.50/0.80 for small/medium/large effects.

## Data Availability

The data presented in this study are available upon request from the corresponding author. The data are not publicly available because they contain information that could compromise the privacy of the participants.

## Supplementary Materials

The following supporting information can be downloaded at: https://www.mdpi.com/article/10.3390/healthcare14213707/s1, File S1: STROBE Statement—checklist of items that should be included in reports of cross-sectional studies; Table S1: Borich needs assessment of home-based end-of-life hospice education items.

## Abbreviations

APN	Advanced practice nurse
ANOVA	Analysis of variance
EoL	End-of-life
HEHC	Home-based end-of-life hospice care
IPA	Importance–performance analysis
IRB	Institutional review board
MCL	Mean required competence level
PCL	Present competence level
RCL	Required competence level
STROBE	STrengthening the Reporting of OBservational studies in Epidemiology
