# Supplementary material for: Educational Needs for Home-Based End-of-Life Hospice Care Among Community Home-Visiting Nurses
Source: Healthcare (Basel). 2026 Nov 1;14(21):3707. doi: 10.3390/healthcare14213707 (PMC13763510; doi:10.3390/healthcare14213707)
Supplement: Supplementary file 1 [file healthcare-14-03707-s001.zip › SUBMIT_Supplementary_File_S1_STROBE_checklist.pdf]

Supplementary Material

S1. STROBE Statement — checklist of items that should be included in reports of cross-sectional studies

Manuscript: Educational Needs for Home-Based End-of-Life Hospice Care Among Community Home Visiting Nurses. Section numbers refer to the revised manuscript.

Section/topic	Item	Recommendation	Location in manuscript	Status
Title and abstract	1 (a)	Study design indicated with a commonly used term in the title or the abstract	Abstract ("descriptive cross-sectional study")	Reported
	1 (b)	Informative and balanced summary of what was done and what was found, in the abstract	Abstract	Reported
Introduction	2	Background/rationale: scientific background and rationale for the investigation	Section 1	Reported
	3	Objectives, including any prespecified hypotheses	Section 1 (final paragraph)	Reported
Methods	4	Study design: key elements presented early in the paper	Section 2.1	Reported
	5	Setting: setting, locations and relevant dates, including periods of recruitment and data collection	Section 2.2 (Chungcheong region, Republic of Korea); Section 2.4 (9–26 July 2026)	Reported
	6 (a)	Participants: eligibility criteria and the sources and methods of selection of participants	Section 2.2	Reported
	7	Variables: all outcomes, exposures, predictors, potential confounders and effect modifiers clearly defined	Sections 2.3.1 and 2.3.2	Reported
	8	Data sources/measurement: sources of data and details of methods of assessment for each variable	Section 2.3.2 (instrument adaptation, I-CVI/S-CVI, Cronbach's α)	Reported
	9	Bias: any efforts to address potential sources of bias	Section 2.5 (Bonferroni correction for the 56 item-level comparisons); Section 4.1 (convenience sampling; self-reported performance; exclusion of non-completers; mean imputation of 0.13% of responses). No a priori design-stage measures to control bias were undertaken.	Partly reported
	10	Study size: how the study size was arrived at	Section 2.2 (G*Power 3.1; $\alpha = 0.05$, $d = 0.25$, power = 0.80; minimum n = 128; 120 analyzed)	Reported
	11	Quantitative variables: how quantitative variables were handled; groupings chosen and why	Section 2.3.1 (experience variables grouped to reflect career stages relevant to the design of staged educational programs); category boundaries shown in Tables 1 and 2. The rationale for the age bands is not stated.	Partly reported
	12 (a)	Statistical methods, including those used to control for confounding	Section 2.5	Reported
	12 (b)	Methods used to examine subgroups and interactions	Section 2.5 (independent t-tests and one-way ANOVA with Scheffé post hoc tests across participant characteristics; Levene's test for homogeneity of variance). Interactions were not examined.	Partly reported
	12 (c)	How missing data were addressed	Section 2.5. Of the 128 questionnaires submitted, eight from respondents who left 20% or more of the instrument unanswered were excluded as non-completers, and 120 were analyzed. The 18 remaining item-level missing responses (0.13%) were replaced with the arithmetic mean of the corresponding item across participants (mean imputation). A sensitivity analysis restricted to complete cases (n = 107) is	Reported

			reported in Section 3.5.	
	12 (d)	If applicable, how the sampling strategy was taken into account in the analysis	Section 2.2 (convenience sample; no design weights applied)	Reported
	12 (e)	Any sensitivity analyses	Section 2.5 and Table 2 note (Welch's ANOVA with Games–Howell post hoc tests where Levene's test indicated unequal variances).	Reported
Results	13 (a)	Numbers of individuals at each stage of the study	Section 2.2 (171 accessed the survey; 128 submitted a questionnaire, submission rate 74.9%; eight excluded as non-completers; 120 analyzed)	Reported
	13 (b)	Reasons for non-participation at each stage	Not reported.	Not reported
	13 (c)	Consider use of a flow diagram	Not included. Participant flow is described in text (Section 2.2).	Not reported
	14 (a)	Descriptive data: characteristics of study participants and information on exposures and potential confounders	Section 3.1 and Table 1	Reported
	14 (b)	Number of participants with missing data for each variable of interest	Section 2.5 (number and proportion of imputed item-level responses, and the number of items affected). The 18 omissions were scattered across 16 of the 112 item ratings, with no more than two in any single item.	Reported
	15	Outcome data: report numbers of outcome events or summary measures	Sections 3.3–3.5; Table 3 (domain level), Table S1 (item level, Supplementary Material), Figure 1	Reported
	16 (a)	Main results: unadjusted estimates and their precision (e.g., 95% confidence interval)	Sections 3.2–3.5; Tables 2 and 3 and Table S1. Means, standard deviations, t and F statistics and p values are reported throughout. Effect sizes are reported for every comparison (Cohen's d or η^2 in Table 2; Cohen's d z in Table 3 and Table S1), and 95% confidence intervals for the importance–performance gap are reported by domain in Table 3.	Reported
	16 (b)	Report category boundaries when continuous variables were categorized	Table 2; Table 1	Reported
	16 (c)	If relevant, translate estimates of relative risk into absolute risk	Not applicable; no relative risk estimates were computed.	Not applicable
	17	Other analyses: analyses of subgroups and interactions, and sensitivity analyses	Section 3.2 (subgroup comparisons by participant characteristics); Sections 3.4–3.5 (Borich needs ranking combined with IPA quadrants); Table 2 note (Welch's ANOVA and Games–Howell tests where variances were unequal). No interaction analyses were performed.	Partly reported
Discussion	18	Key results: summarise key results with reference to study objectives	Section 4 (opening paragraphs)	Reported
	19	Limitations: limitations of the study, taking into account sources of potential bias or imprecision; direction and magnitude of any potential bias	Section 4.1	Reported
	20	Interpretation: cautious overall interpretation considering objectives, limitations, multiplicity of analyses, results from similar studies and other relevant evidence	Section 4; Section 5	Reported
	21	Generalisability: generalisability (external validity) of the study results	Section 4.1 (convenience sample from one region; 95.8% women)	Reported
Other information	22	Funding: source of funding and the role of the funders for the present study	Funding statement	Reported

Note: Recommendations are summarised. The official STROBE checklist for cross-sectional studies (version 4) is available at https://www.strobe-statement.org, and this completed form follows its item numbering.

The limitations section (Section 4.1) covers sampling and generalisability, the use of self-reported performance, and the cross-sectional design. Items marked “Not reported” were not undertaken in this study. STROBE is an explanatory tool; items not applicable to this descriptive cross-sectional design are marked accordingly.
