# Supplementary material for: Educational Needs for Home-Based End-of-Life Hospice Care Among Community Home-Visiting Nurses
Source: Healthcare (Basel). 2026 Nov 1;14(21):3707. doi: 10.3390/healthcare14213707 (PMC13763510; doi:10.3390/healthcare14213707)
Supplement: Supplementary file 1 [file healthcare-14-03707-s001.zip › SUBMIT_Supplementary_Table_S1_Borich_needs.pdf]

Supplementary Material S2. Table S1. Borich needs assessment of home-based end-of-life hospice education items (N = 120).

Domain	No.	Item	Importance $M \pm$ SD	Performance $M \pm$ SD	Gap	t	p	d_z	Borich needs	Rank	IPA quadrant
A	1	Philosophical and stage-based understanding of death	4.34 ± 0.72	3.12 ± 1.06	1.225	10.485	<0.001	0.96	5.319	11	II
	2	Medical understanding of death: physical changes in terminally ill patients and explanation of signs of impending death	4.33 ± 0.75	3.31 ± 1.12	1.017	7.340	<0.001	0.67	4.397	40	IV
	3	Understanding psychological responses of families of patients at the end of life	4.38 ± 0.71	3.34 ± 1.13	1.042	8.124	<0.001	0.74	4.566	39	I
	4	Meaning of death and attitudes and responses toward death	4.35 ± 0.78	3.30 ± 1.07	1.053	8.374	<0.001	0.76	4.583	37	I
B	5	Understanding the philosophy and goals of hospice and palliative care	4.29 ± 0.71	3.16 ± 1.22	1.132	9.299	<0.001	0.85	4.858	28	III
	6	Understanding differences between home and hospital hospice	4.33 ± 0.70	3.13 ± 1.23	1.200	8.551	<0.001	0.78	5.200	17	III
	7	Multidisciplinary teams in hospice and palliative care	4.20 ± 0.73	2.96 ± 1.18	1.242	8.899	<0.001	0.81	5.215	16	III
	8	Current status and future directions of hospice and palliative care in Korea and abroad	4.13 ± 0.83	2.83 ± 1.17	1.308	9.630	<0.001	0.88	5.408	9	III
C	9	Life-sustaining treatment systems and advance care planning	4.42 ± 0.73	3.25 ± 0.98	1.167	9.698	<0.001	0.89	5.153	18	I
	10	Decision-making related to withdrawal of life-sustaining treatment	4.50 ± 0.64	3.22 ± 1.05	1.283	10.780	<0.001	0.98	5.775	5	II
	11	Management of situations involving ethical dilemma	4.40 ± 0.67	3.20 ± 1.11	1.200	9.195	<0.001	0.84	5.280	12	II
	12	Ethical basis for truth-telling	4.18 ± 0.71	3.09 ± 1.11	1.093	8.133	<0.001	0.74	4.575	38	III
	13	Protection of privacy and dignity in the home setting	4.28 ± 0.83	3.27 ± 1.05	1.008	7.928	<0.001	0.72	4.319	42	IV
	14	Legal issues, including wills, inheritance management, and organ transplantation	4.34 ± 0.67	2.92 ± 1.18	1.426	10.544	<0.001	0.96	6.193	3	II
D	15	Understanding pain and pharmacological and non-pharmacological treatment	4.52 ± 0.72	3.45 ± 1.11	1.067	8.980	<0.001	0.82	4.818	29	I
	16	Management of respiratory and circulatory symptoms	4.57 ± 0.68	3.42 ± 1.12	1.150	9.545	<0.001	0.87	5.252	13	I
	17	Management of gastrointestinal symptoms	4.47 ± 0.65	3.35 ± 1.07	1.117	9.157	<0.001	0.84	4.988	24	I
	18	Management of pressure injuries and skin damage	4.42 ± 0.76	3.59 ± 1.05	0.833	6.797	<0.001	0.62	3.687	53	I

	19	Management of neuropsychiatric symptoms and palliative sedation	4.38 ± 0.70	3.16 ± 1.14	1.225	9.718	<0.001	0.89	5.370	10	II
	20	Management of other symptoms, including emergencies, neurological and endocrine symptoms, and sleep disorders	4.45 ± 0.62	3.31 ± 1.04	1.142	9.730	<0.001	0.89	5.080	20	I
	21	Assessment and evaluation of terminal status	4.52 ± 0.61	3.38 ± 1.12	1.139	9.817	<0.001	0.90	5.142	19	I
	22	Nutritional management	4.33 ± 0.74	3.37 ± 1.03	0.955	7.512	<0.001	0.69	4.131	45	IV
E	23	Guided imagery and expressive therapies, including meditation, art, music, and aromatherapy	3.98 ± 0.77	2.67 ± 1.15	1.317	10.375	<0.001	0.95	5.245	14	III
	24	Manipulative and body-based therapies, including massage and acupressure	4.07 ± 0.88	2.71 ± 1.05	1.361	11.274	<0.001	1.03	5.534	7	III
F	25	Criteria for emergency transport via 119	4.46 ± 0.68	3.37 ± 1.11	1.092	9.179	<0.001	0.84	4.867	27	I
	26	Understanding criteria for referral to a physician or emergency department	4.42 ± 0.68	3.35 ± 1.14	1.075	8.775	<0.001	0.80	4.757	32	I
	27	Response to home hospice emergencies, including dyspnea, delirium, bleeding, and catheter drainage problems	4.43 ± 0.72	3.37 ± 1.19	1.067	8.006	<0.001	0.73	4.729	34	I
G	28	Assessment and evaluation of psychosocial needs	4.16 ± 0.72	3.23 ± 1.08	0.933	7.529	<0.001	0.69	3.881	50	III
	29	Psychosocial and socioeconomic care strategies	4.28 ± 0.67	3.16 ± 1.12	1.117	8.949	<0.001	0.82	4.774	31	III
	30	Understanding spirituality and spiritual needs	4.22 ± 0.66	3.04 ± 1.20	1.177	9.685	<0.001	0.88	4.964	25	III
	31	Spiritual care	4.25 ± 0.71	3.02 ± 1.22	1.233	10.435	<0.001	0.95	5.242	15	III
	32	Empathic communication skills	4.23 ± 0.80	3.41 ± 1.05	0.825	6.294	<0.001	0.57	3.493	55	IV
	33	Communication considering cultural and religious factors	4.18 ± 0.71	3.26 ± 1.05	0.925	7.733	<0.001	0.71	3.870	52	IV
	34	Management of anxiety and depression in patients and families	4.39 ± 0.73	3.51 ± 1.06	0.883	6.753	<0.001	0.62	3.879	51	I
	35	Understanding the dying experience and care before and after death	4.52 ± 0.71	3.40 ± 1.19	1.117	8.716	<0.001	0.80	5.044	23	I
H	36	Assessment of family caregiver burden	4.46 ± 0.70	3.39 ± 1.05	1.063	8.878	<0.001	0.81	4.741	33	I
	37	Support and education for caregivers	4.47 ± 0.66	3.43 ± 1.03	1.033	8.565	<0.001	0.78	4.616	36	I
	38	Understanding grief and bereavement	4.33 ± 0.72	3.37 ± 1.09	0.958	7.380	<0.001	0.67	4.145	44	IV
	39	Assessment and intervention for caregiver stress and burnout	4.35 ± 0.76	3.25 ± 1.07	1.100	8.334	<0.001	0.76	4.785	30	I

I	40	Documentation and information management, including electronic nursing records	4.42 ± 0.61	3.37 ± 1.04	1.054	9.718	<0.001	0.89	4.657	35	I
	41	Financial management, including reimbursement and health insurance	4.33 ± 0.71	3.16 ± 1.20	1.167	9.301	<0.001	0.85	5.046	21	III
	42	Safety guidelines and risk management for solo home visits	4.32 ± 0.71	3.35 ± 1.09	0.967	7.755	<0.001	0.71	4.173	43	IV
J	43	Use of personal protective equipment and disinfection of equipment during home visits	4.23 ± 0.82	3.48 ± 1.04	0.758	5.731	<0.001	0.52	3.210	56	IV
	44	Precautions when visiting patients with infectious diseases	4.44 ± 0.72	3.53 ± 0.99	0.908	7.477	<0.001	0.68	4.032	48	I
	45	Assessment of home environmental hazards, including falls, oxygen, electricity, pressure injury risks, and hygiene	4.54 ± 0.62	3.64 ± 1.03	0.899	7.539	<0.001	0.69	4.080	47	I
K	46	Understanding and caring for patients with non-cancer terminal illnesses	4.43 ± 0.63	3.53 ± 1.06	0.900	7.746	<0.001	0.71	3.990	49	I
	47	Understanding and caring for older adults	4.45 ± 0.70	3.64 ± 0.98	0.808	7.484	<0.001	0.68	3.597	54	I
L	48	Children's understanding of death	4.28 ± 0.77	2.72 ± 1.15	1.558	11.694	<0.001	1.07	6.662	1	III
	49	Care methods for children and their families	4.19 ± 0.79	2.67 ± 1.13	1.525	11.966	<0.001	1.09	6.392	2	III
M	50	Stress and burnout management	4.43 ± 0.69	3.17 ± 1.13	1.267	11.055	<0.001	1.01	5.616	6	II
	51	Leadership development	4.23 ± 0.75	3.21 ± 1.20	1.025	8.316	<0.001	0.76	4.339	41	III
	52	Continuing education	4.28 ± 0.74	3.32 ± 1.09	0.964	8.027	<0.001	0.73	4.129	46	IV
N	53	Digital-based home hospice care, including remote monitoring, counseling, and mobile-app-based symptom management	4.17 ± 0.77	2.97 ± 1.17	1.208	8.824	<0.001	0.81	5.045	22	III
	54	Current status and future directions of digital hospice and palliative care in Korea and abroad	4.14 ± 0.78	2.96 ± 1.25	1.183	8.543	<0.001	0.78	4.901	26	III
O	55	Strategies for linkage and partnership with community and external institutions	4.39 ± 0.66	3.01 ± 1.17	1.378	11.387	<0.001	1.04	6.046	4	II
	56	Partnership with professionals from other disciplines	4.37 ± 0.65	3.12 ± 1.18	1.253	10.149	<0.001	0.93	5.476	8	II

Gap = importance − performance. All paired comparisons were significant at $p < 0.001$ after Bonferroni correction (adjusted $\alpha = 0.0009$). d_z = Cohen's d for paired samples ($t/\sqrt{N}$). Borich needs score = Σ[(RCL − PCL) × MCL]/N. IPA Quadrant I = keep up the good work (high importance/high performance); II = concentrate here (high importance/low performance); III = low priority (low importance/low performance); IV = possible overkill (low importance/high performance). Quadrants were defined using the grand means of importance (4.34) and performance (3.23). The domain letters correspond to those in Table 3.
